# Estimating Pavement Roughness and Macrotexture Using Vehicles Equipped with Smart Tires

**DOI:** 10.3390/s26144565

**Published:** 2026-07-18

**Authors:** Aliasghar Akbari Nasrekani, Lucia Tsantilis, Davide Dalmazzo, Davide Chiola, Riccardo Ricci, Benedetto Carambia, Ezio Santagata

**Affiliations:** 1 Department of Environment, Land and Infrastructure Engineering (DIATI), Politecnico di Torino, Corso Duca degli Abruzzi 24, 10129 Turin, Italy; lucia.tsantilis@polito.it (L.T.); davide.dalmazzo@polito.it (D.D.); 2 Research and Development Department, Movyon S.p.A., Via Alberto Bergamini 50, 00159 Rome, Italy; davide.chiola@movyon.com (D.C.); riccardo.ricci@movyon.com (R.R.); benedetto.carambia@movyon.com (B.C.); 3 Department of Civil and Environmental Engineering, Qatar University, Doha 2713, Qatar; ezio.santagata@qu.edu.qa

**Keywords:** smart tires, pavement roughness, pavement macrotexture, Dynamic Index (DI), Pr index, international roughness index (IRI), mean profile depth (MPD)

## Abstract

In the context of pavement management, conventional data collection methods for the evaluation of pavement functional condition are limited by relatively slow acquisition speeds, that prevent fast-lane motorway surveying at 120–130 km/h, and by survey frequency, which on vast networks typically occurs twice a year. Given these limitations, continuous pavement condition monitoring from moving vehicles offers an attractive solution to move towards real-time digital road assessment. In particular, such a result is achieved by making use of “intelligent” or “smart” tires, which by means of appropriate arrays of sensors can capture contact patch information, thereby providing quantitative information related to pavement roughness and macrotexture. In this study, smart tire data functional condition indicators, Dynamic Index (DI) and Pr index, were collected over several segments of a motorway network, with a total length of 405 km. Correlations were investigated between such parameters and the results of measurements coming from a traditional pavement monitoring technique, expressed in terms of international roughness index (IRI) and mean profile depth (MPD). Furthermore, the ability of smart tire indicators to identify time-dependent trends and to rank different motorway segments was assessed. Obtained results, which were generated by adopting different data processing and homogenization techniques, showed that DI displays a moderate correlation with IRI, while Pr exhibits a strong correlation with MPD. Pavement-age analysis highlighted the existence of meaningful trends for both dense-graded and open-graded asphalt-wearing courses. Motorway rankings based on average DI and Pr values were found to be in agreement with those obtained from average IRI and MPD values, thereby confirming the potential of smart tire technology as a complementary network-level monitoring tool for pavement asset management systems.

## 1. Introduction

Road pavements in many countries worldwide deteriorate more rapidly than expected, mainly as a result of the exceedingly high and unpredicted growth of traffic volumes and loads, and because of uncertain changes in climate and environmental conditions. These factors accelerate both structural and functional damage, causing pavements to reach unsatisfactory conditions before the end of their design lives. Thus, if timely interventions are not carried out within the intended service life, road agencies are forced into major rehabilitation or full reconstruction, which are far more expensive than planned preventive interventions or minor corrective works [1].

To address these challenges, many road agencies have adopted pavement asset management systems (PAMS) to assist them in maintaining deteriorating pavement assets in good conditions. However, these systems can be beneficial only if they are based on a truly representative, accurate and frequently updated database, which is considered the heart of any PAMS. In other words, the soundness of decisions generated by a PAMS is strongly influenced by how and when data are collected, and by the analytical methods used for their processing [1,2].

The present study narrows down this broad context to the functional condition assessment of roads, expressed in terms of pavement roughness and macrotexture. These pavement features are the key elements of most available PAMSs in describing the current state of the road network, in supporting the forecast of future conditions and in consequently selecting the most appropriate maintenance or rehabilitation strategies. The collection of functional condition data is generally performed by means of fully equipped survey vehicles (e.g., ARAN, Pathrunner, MFV, ROMDAS, NSV, Laser RST) carrying cameras, lasers, accelerometers, and other sensors which can capture roughness, texture and surface distresses while traveling at normal traffic speeds. Although these vehicles are employed worldwide, for the inspection of extended motorway networks they are affected by limitations which mainly derive from the relatively slow speed of data collection (that cannot reach 120–130 km/h). In particular, fast lanes cannot be easily subjected to assessment, and the overall network can rarely be surveyed more than twice a year. Thus, the resulting functional condition database is not sufficiently representative and does not maximize the efficiency of the available PAMS.

To overcome the deficiencies outlined above, road agencies may consider moving towards real-time digital monitoring by means of alternative strategies, which entail the continuous collection of pavement condition data from moving vehicles. To this end, one interesting solution is to convert tires from passive to active elements, implementing “intelligent” or “smart” features that allow the acquisition of contact patch information pertaining to the roughness, texture, and presence of surface distresses.

## 2. Literature Review on Smart Tires

After being introduced by Pohl et al. [3], the concept of “intelligent” or “smart” tires has gained significant interest among researchers, and over the past two decades, both industry and academia have proposed and tested various architectures while addressing a multitude of challenging tasks [4,5]. For example, Braghin et al. [6] carried out a preliminary study in which triaxial accelerometers were identified as the most appropriate sensors to be used in tires. However, various other sensing technologies have been explored for the development of intelligent tires. These include strain sensors, surface acoustic wave (SAW) sensors, piezoelectric sensors combined with energy harvesting elements, and optical sensors, as investigated by different researchers [7,8].

Since the automotive industry may gain the greatest benefits from the development of smart tires—via improving vehicle control, advanced driver assistance and driver information systems—most research on this specific technology so far has focused on applications related to vehicles and robotic prototypes [4,9,10,11,12,13,14,15,16,17,18,19,20,21,22,23].

Apart from the automotive industry—which is not the focus sector of the research described in this paper—road authorities can also benefit greatly from the development and widespread use of intelligent tires. In such a context, road condition recognition using such a technology has been approached mainly by treating the tire as a vibration sensor that “feels” the surface, and by extracting features from in-tire signals to infer surface type, roughness, or friction.

Early work by Braghin et al. [6] focused on sensor placement and basic roughness sensing. In the performed investigation, triaxial accelerometers were mounted on the inner liner at the center and shoulders of the tread, since this location was deemed adequate to protect the sensors from external damage while avoiding changes to local stiffness. With wired power and data transmission, and a 10 kHz sampling rate, when referring to measurements executed on a test track, it was found that the filtered root mean square (RMS) value of radial acceleration, computed when the sensor was out of contact, increased systematically from smooth to rough asphalt and depended mainly on texture and speed (and only weakly on tire size). This outcome indicated that a smart tire, once calibrated, can be used as a road texture sensor.

Subsequent studies exploited the frequency-domain characteristics of acceleration signals to classify surface and friction conditions more explicitly. Morinaga et al. [24] used longitudinal acceleration at the tread center, thereby showing that high-frequency components (>2 kHz) in the contact patch were characteristic of low-friction ice, while high-frequency peaks ahead of the contact patch indicated the presence of water films on the road surface. By designing band-pass filters around these bands, road surfaces could be ultimately classified as dry, wet, icy, or snowy. Singh et al. [4] measured circumferential acceleration near the contact patch on different asphalt textures, splitting each revolution into pre- and post-trailing domains. Obtained results showed that most pavement surface information lay in the pre-trailing part. By defining low- and high-frequency bands and using the ratio of high-band to low-band power as an indicator, this ratio was combined with tire speed and pressure in a fuzzy-logic classifier that distinguished smooth, regular, rough, and wet asphalt with about 90% accuracy under low-slip conditions.

Another line of work focused on friction potential and contact geometry. Niskanen and Tuononen [19] used multiple three-axis accelerometers inside a car tire on ice and concrete at various speeds and pressures, showing that contact length, derived from longitudinal acceleration peaks at leading and trailing edges, decreased with increasing pressure and could act as a friction indicator independent of surface roughness. Further analyses considered high-frequency acceleration just ahead of the contact patch, using band-pass filtering (in the 2–5 kHz range) and spectral energy as a friction proxy, showing that higher energy was observed on ice compared to concrete, consistent with more local slip. Based on obtained results, it was concluded that a single three-axis accelerometer placed behind the central tread ribs was sufficient to capture both contact length and friction-related vibrations.

The same basic principles have been extended to robotic and classification applications. Khaleghian and Taheri [22] instrumented a six-wheel ground robot with a triaxial in-tire accelerometer and wheel-speed encoders, and used low-frequency radial acceleration power (<20 Hz) together with initial slip ratio to build a fuzzy-logic terrain classifier distinguishing asphalt, concrete, soil, and grass. Recorded results showed that harder surfaces produced higher radial vibration power and lower initial slip, while softer surfaces damped vibrations and induced higher slip, enabling robust surface recognition across speeds. In the vehicle domain, Kim et al. [5] combined an in-tire three-axis accelerometer, FFT-based spectral features, and a small feed-forward neural network to classify dry, wet, and rough asphalt. Accuracies above 98% for dry and 100% for wet and rough conditions were reported, suggesting that relatively simple neural networks can be sufficient for basic surface classification.

More recent studies have extended the scope from discrete condition labels to pavement roughness indices. Montorio et al. [25] used radial acceleration from intelligent tires to define a deformation-based index (Dynamic Index, DI) in the wavelength band relevant to the international roughness index (IRI). DI, computed from in-tire deformation signals, was correlated with IRI values obtained from a reference profiler over multiple highway sections, with a reported R squared of 0.57, thereby indicating that vehicle fleets equipped with smart tires can support network-level IRI monitoring. In parallel, several other works emphasized the potential benefits deriving from the integration of smart tires and machine learning. Lee et al. [26] trained fully connected and convolutional networks directly on multi-axis in-tire accelerations for road surface classification, showing that convolutional models can classify multiple pavement types in real time. Han et al. [27] used a compact time-series model on similar data to estimate adhesion across speeds. Xia et al. followed a feature-engineering approach, denoising and segmenting circumferential acceleration per revolution, extracting statistical and wavelet-based features, applying principal component analysis, and then training a small neural network to classify low-, medium-, and high-friction pavements with very high accuracy [28].

Finally, other authors have shown that alternative in-tire sensors can support road condition recognition and roughness estimation. Dynamic tire pressure sensors combined with axle accelerometers have been used to infer IRI from dynamic pressure changes, achieving results comparable to laser profilers on expressways [29,30,31], while strain-based piezoelectric cables bonded to the inner liner and sidewall have been used to classify roads into roughness levels and to detect anomalies via machine-learning models [32]. Dózsa et al. used a piezoresistive force sensor embedded in the tire, and time–frequency analysis of its signals to extract threshold-based and neural classifiers, demonstrating that simple time–frequency features provide strong correlations with empirical road-quality classes [33].

## 3. Research Motivation and Goal

Previous studies demonstrated that intelligent tires, equipped with accelerometers, pressure sensors, or strain sensors, can capture characteristic road-induced signals that, after suitable processing or machine-learning analysis, allow road surface classification, friction estimation, and roughness assessment for use in infrastructure monitoring. However, most existing systems still rely on wired connections, low or medium test speeds, and small sets of very distinct surface classes, which limit their suitability for mass production, real-traffic speeds, and the detailed assessment of pavement texture and roughness. In particular, most of the investigated systems do not provide results expressed in terms of standardized metrics, and have been validated only on short road sections with narrow IRI ranges, remaining largely untested over extended networks in varied conditions.

In response to the above, the research study described in this paper aims to highlight the full potential of the smart tire technology by considering data collected along the component segments of a vast motorway network, and by investigating the relationships existing between parameters drawn from their analysis and the results obtained by means of traditional surveying techniques. The ultimate goal is to verify whether the use of these tires represents a reliable and scalable solution to measure asphalt pavement roughness and texture at relatively high operating speeds, delivering frequent and lane-specific measurements that can be fed to pavement management systems.

It should be emphasized that the novelty of this study does not lie in the original definition of the Dynamic Index and Pr index, which were introduced in previous research, but in their large-scale validation and practical interpretation for motorway pavement management. Furthermore, this study provides, for the first time in the scientific literature, a comprehensive validation of a wireless smart tire-based methodology for high-speed pavement texture assessment against standardized ARAN-derived MPD measurements using an extensive 405 km motorway dataset collected under real traffic conditions. Compared with previous studies, it evaluates both roughness- and macrotexture-related tire indicators and demonstrates their applicability to homogeneous-section analysis, pavement-age interpretation, and network-level pavement ranking within PAMS.

## 4. Pavement Roughness and Pavement Texture

Pavement surface texture is defined by referring to the deviations in the pavement surface from a true planar surface [34]. These deviations occur at three distinct scale levels, each defined by the wavelength (λ) and peak-to-peak amplitude (A) of its components. The three levels of texture, as established by the Permanent International Association of Road Congresses (PIARC) [35], are as follows:

- Microtexture (λ < 0.5 mm, A = 1 to 500 μm): Surface roughness at the sub-visible or microscopic level, which is a function of the surface texture properties of the aggregate particles contained in the asphalt- or concrete-paving material;
- Macrotexture (λ = 0.5 to 50 mm, A = 0.1 to 20 mm): Surface roughness defined by the overall properties (shape, size, and gradation of aggregates) of asphalt-paving mixtures, or by the method of finishing and texturing (dragging, tining, grooving; depth, width, spacing and orientation of channels/grooves) used on a concrete-paved surface;
- Megatexture (λ = 50 to 500 mm, A = 0.1 to 50 mm): Texture with wavelengths in the same order of size as the pavement–tire interface, largely defined by the distresses and defects present on the pavement surface.

Wavelengths greater than the upper limit (500 mm) of megatexture are associated with what is defined as roughness or unevenness [36]. From the viewpoint of the road user, rough roads lead to discomfort, decreased speed, potential vehicle damage, and increased operating costs. Therefore, roughness is a condition indicator that should be carefully considered when evaluating primary pavements for safety and serviceability [37].

Macrotexture plays a critical role in tire–pavement friction by controlling the hysteresis component of friction force created by tire rubber deformation, which becomes increasingly important on wet surfaces and at higher speeds. Macrotexture also governs surface drainage by providing escape channels for water in the contact patch, thereby reducing aquaplaning and water splash and helping to maintain effective tire–pavement contact. For this reason, it is regarded as a key functional condition indicator when evaluating primary pavements for safety and serviceability [34,37].

Both pavement roughness and macrotexture evolve in time as a function of environmental exposure and traffic loading. In particular, previous studies have shown that roughness can progressively increase depending upon differential settlements in unbound layers and subgrade, as well as the result of the post-construction uneven densification of asphalt mixtures. On the other hand, macrotexture evolution can be described by referring to the simultaneous effects of several phenomena, which include aggregate polishing and bleeding (which tend to reduce macrotexture) and surface raveling (which has the opposite effect, causing a localized increase in macrotexture). Given the complexity of the scenarios associated with the time-dependent changes in roughness and macrotexture, a multitude of descriptive and predictive models have been formulated by researchers, who have lately been focusing on the use of machine learning algorithms [38,39,40].

In the following, the two standard methods typically employed for measuring pavement roughness and macrotexture are illustrated.

### 4.1. International Roughness Index (IRI)

The international roughness index (IRI) is a standardized profile-based measure of pavement roughness introduced by the World Bank after the 1982 International Road Roughness Experiment to provide a common reference scale for correlating different roughness measurement systems. It is defined as the response of a standardized quarter-car model traveling at 80 km/h over a measured longitudinal profile, expressed in m/km or in/mi as the accumulated suspension motion per unit distance [41,42].

In practice, IRI is computed by feeding a sequence of uniformly spaced elevation points along each wheel path into the quarter-car algorithm, with agencies typically following the procedures specified in ASTM E1926, AASHTO R43, or EN 13036-5 [42,43,44]. An IRI of 0 m/km represents a perfectly smooth profile, whereas values above 8 m/km correspond to pavements that are nearly impassable at normal speeds. As per pavement engineering practice, intermediate thresholds are used to classify pavement conditions for construction quality control and network-level management. Standard implementation methodologies accept ASCII-formatted profile files (e.g., Fortran 1X,F8.3,1X,F8.3), optionally pre-filtered with a 250 mm moving average to account for tire-contact effects, before simulating the quarter-car response to obtain the final IRI value [42,45].

### 4.2. Mean Profile Depth (MPD)

The mean profile depth (MPD) is defined as the primary profile-based descriptor of pavement macrotexture in ISO 13473-1 and ASTM E1845 [46,47]. It is derived from a two-dimensional longitudinal profile measured by an inertial profilometer with a horizontal sampling interval not exceeding 1 mm, and is intended to represent macrotexture with wavelengths between 0.5 mm and 50 mm, while largely excluding microtexture and long-wavelength unevenness. Invalid readings caused by photometric effects, shadowing, or other artifacts are identified, removed, and interpolated, and the profile is spatially filtered so that components with wavelengths greater than 100 mm and shorter than 2.5 mm are attenuated, leaving mainly the macrotexture band.

MPD is then calculated on 100 mm ± 10 mm baseline units, each divided into two 50 mm halves, by detecting the highest peak in each half and by taking the arithmetic mean of these peak levels relative to the average profile level, which is effectively zero after filtering or slope suppression. Repeating this procedure over the units of a test section yields a set of MPD values, and the overall macrotexture descriptor is reported as their arithmetic mean, together with the standard deviation and the number of contributing units [46,47].

## 5. Research Methodology

In order to fully explore the potential of using smart tires for the evaluation of pavement functional conditions, the research work described in this paper was based on the analysis of data collected along several component segments of a motorway network. In particular, data were available for the six motorway segments listed in Table 1, with a total extension of 405 km. Selected segments were considered to be representative of the entire network, being characterized by varied environmental conditions and traffic volumes.

In the course of one semester, surveys were executed with vehicles equipped with smart tires on a weekly basis (from week 27 to week 52), with multiple passes performed along the slow lane of the same road sections. Reference measurements of IRI (as per ASTM E1926) and MPD (as per BS EN ISO 13473-1) were conducted once over the network by using an automatic road analyzer (ARAN) vehicle.

### 5.1. Smart Tire Data

Some technical specifications of the smart tires and acquisition system are covered by manufacturer confidentiality; however, the parameters that can be disclosed and directly affect the interpretation of experimental data are provided in the following.

The vehicles employed for the pavement surveys were passenger cars equipped with smart tires—developed by a specialized tire manufacturer—which were instrumented with MEMS accelerometers directly glued to the inner liner, which sample acceleration along the three orthogonal directions for the monitoring of tire deformation. Vehicle speed ranged approximately from 80 to 90 km/h. Inside each tire, one sensor was installed and different tire configurations were used, but the methodology which is herein described shows that the DI index is independent of tire type, while the radius of the tire is considered in the calculation process of Pr index. Each vehicle was also equipped with two vehicle radio receivers (VRRs), one for the front axle and another one for the rear axle. Each radio receiver collects sensor signals from two antennas located in the right and left fenders, enabling wireless transmission of tire–road interaction signals to the on-board vehicle elaboration unit (VEU) employed for preliminary data processing. All collected data were georeferenced by means of a GPS system which had a sampling rate of 1 Hz with an accuracy of almost 1.5 m. A schematic representation of the smart tire data collection system can be found in the study published by Montorio et al. [25].

Of the three acceleration signals, the radial (vertical) component is selected for analysis due to its greater sensitivity to tire–road interactions. Processing of the data is thereafter carried out according to the proprietary algorithms and procedures developed by the tire manufacturer, synthesized in the following, which leads to the calculation of two characteristic indicators: the Dynamic Index (DI), which is an indicator of pavement roughness, and the so-called Pr index, which is an indicator of pavement macrotexture.

#### 5.1.1. Dynamic Index (DI)

For the calculation of the Dynamic Index (DI), after being filtered, the radial acceleration signal is integrated twice with respect to time to obtain an estimate of radial displacement, which represents the dynamic deformation of the tire as it rolls over the surface. Thereafter, the power spectral density (PSD) value is calculated according to the following equation:

(1) $$PSD=2\times {\left(\frac{1}{F_{s}}\right)}^{2}\times \frac{1}{L_{p}}\times FFT(x)\times conj(FFT\left(x\right)),$$

where $F_{s}$ is the sampling frequency, $L_{p}$ is the length of the period, and FFT(x) is the Fast Fourier Transformation of the tire deformation signal.

The next step of the data processing methodology consists of correlating the information collected from pairs of tires to enhance robustness and filter out random noise. This requires the estimation of the coherence function (Coh) by means of the following equation:

(2) $$Coh=\frac{real\left(2\times {\left(\frac{1}{F_{s}}\right)}^{2}\times \frac{1}{L_{p}}\times FFT(x_{1})\times conj(FFT\left(x_{2}\right))\right)}{{\left({PSD}_{x_{1}}\right)}^{0.5}\times {\left({PSD}_{x_{2}}\right)}^{0.5}},$$

where $x_{1}$ is the deformation signal of the first tire, $x_{2}$ is the deformation signal of the second tire, and PSD is the previously defined power spectral density.

The coherence function takes values comprised between negative one and one, where the maximum value indicates that the two tires contribute synchronously at a given wavelength. The minimum value corresponds to a half-period phase shift between the two tires at that wavelength, which occurs when they exhibit a strongly out-of-phase rolling behavior. A coherence equal to zero implies that at least one of the two signals is null in the corresponding wavelength range, so that this input is effectively filtered out from the analysis. The above-mentioned coherence is computed for each couple of deformation signals for the two axes and the two sides of the vehicle.

The last step of the data processing methodology leads to synthetizing the information into a single value. Firstly, the mean values of coherence and PSD are computed to obtain an equivalent PSD (EqPSD) at vehicle level:

(3) $$EqPSD=\frac{PSD\left(x_{FL}\right)+PSD\left(x_{FR}\right)+PSD\left(x_{RL}\right)+PSD\left(x_{RR}\right)}{4}\times \frac{{Coh}_{S_{L}}+{Coh}_{S_{R}}+{Coh}_{a_{F}}+{Coh}_{a_{R}}}{4},$$

where ${Coh}_{S_{L}}$ is coherence on the left side (between front left and rear left tires), ${Coh}_{S_{R}}$ is coherence on the right side (between front right and rear right tires), ${Coh}_{a_{F}}$ is coherence at front axle (between front left and front right tires), and ${Coh}_{a_{R}}$ is coherence at rear axle (between rear left and rear right tires).

The equivalent PSD represents the energy content for each wavelength, which is associated with multiple tires. It is therefore an estimate of the energy transmitted from the road longitudinal profile to the vehicle body. In a similar way to the methodology of IRI calculation from longitudinal profile, which considers wavelengths in the range from 1.25 m up to 30 m, the final index—namely DI—is calculated as the integral value of the above-mentioned equivalent PSD in the wavelength region that, according to the golden car suspension model, mostly affects vehicle response:

(4) $$DI=\sum_{n=1/30}^{1/1.25}{EqPSD}_{n},$$

It should be mentioned that IRI values are commonly estimated separately for each side of the vehicle by considering different longitudinal profiles for the left and right wheel track. Consequently, for comparative reasons, the DI index can also be estimated based on the deformation signals coming from the pairs of tires on the same side of the vehicle. According to this additional approach, the equivalent PSD is calculated as the mean value of the PSD of the two tires times the coherence between them:

(5) $${EqPSD}_{L}=\frac{PSD\left(x_{FL}\right)+PSD(x_{RL})}{2}\times {Coh}_{S_{L}},$$

As a result of the above, in addition to the previously defined “vehicle DI”, two more indexes are defined, namely “DI left” and “DI right”, which can be related to the IRI obtained for the left and right wheel track, respectively [25].

#### 5.1.2. Pr Index

The procedure that is followed for the calculation of the Pr index is currently covered by an international patent [48]. In such a context, it should be specified that the present study does not propose a new Pr formulation but evaluates the field response of the available Pr index against the standardized MPD parameter over an extended motorway network.

According to the information shared by the tire manufacturer, it is based on the processing of radial acceleration signals which eventually leads to an index which expresses the degree of macrotexture similarity that the inspected pavement exhibits in comparison to selected reference pavement surfaces. These are identified as rough (with cobblestones) and smooth (finely textured asphalt). However, rather than providing a discrete label classification, the Pr index is a continuous metric computed on a per-wheel-turn basis.

Following data collection and processing, four possible outcomes and corresponding interpretations are obtained:

Pr < 0: Smoother than smooth reference

0 < Pr < 1: Between smooth and rough

1 < Pr < 2: Between rough and reference cobblestone

Pr > 2: Rougher than reference cobblestone

### 5.2. Creation of Network Database

For the purpose of the research project, a comprehensive database of the motorway network was created by merging together all available and relevant information, ensuring full compatibility between data coming from different sources.

The first step in constructing the database was to examine the data collected during the weekly smart tire surveys and identify the most representative survey for the subsequent analyses. Preliminary analyses showed no appreciable changes in pavement condition during the study period. Consequently, selecting a single representative survey was considered appropriate for evaluating the relationship between the proposed smart tire indicators and the reference pavement condition indices. Week 48 was selected because it offered the most complete coverage of the monitored network, including all five motorways, and contained the smallest number of outliers. In addition, it was necessary to ensure that no maintenance interventions or surface treatments had been carried out between the smart tire surveys and the reference pavement inspections, as such interventions would have compromised the validity of the comparisons.

In addition to the elimination of outliers, preliminary data cleaning required IRI and MPD data (available at 20 m and 10 m intervals, respectively) to be aligned to DI and Pr data coming from smart tires (available at 20 m intervals), matching the closest relative chainage on the same motorway segment and direction. Furthermore, for the purpose of comparing the two sets of data, only slow-lane DI and Pr values were retained in the database, excluding data that were collected during unavoidable lane changes. Additional information introduced in the database was extracted from the PAMS adopted by the road agency, in which data were available with respect to the type and age of the pavement-wearing course, as well as to the presence of bridges and viaducts along the investigated motorway segments.

The network database resulting from the preparatory work outlined above consisted of approximately 20,275 strings (or rows) of data referring to elementary road sections of 20 m length. Preliminary analysis revealed no direct point-to-point correlations between DI and IRI or between Pr and MPD. Consequently, data points were aggregated into homogeneous road sections by using the two alternative methods described in the following.

When following the first method (hereinafter indicated as “Method A”), motorway segments were divided into statistically homogeneous road sections by iteratively verifying the internal consistency of DI and Pr data. Rows with a vehicle DI greater than 2.5 were first removed from the database as outliers. Then, the first 300 m (i.e., the first 15 data points at 20 m spacing) were taken as the starting (or seed) reference section and its mean DI and standard deviation were calculated. When evaluating the adjacent 300 m section, its mean and standard deviation were compared to those of the previous reference section, thereby considering the two sections to be homogeneous if the percentage difference between the pairs of statistical parameters was within 30% and 70% for the mean and standard deviation, respectively. If considered homogeneous, the two elementary sections were merged, with the consequent creation of a new reference section of 600 m length. When the homogeneity criteria were not satisfied, the section coming from the previous step of analysis was closed, and the adjacent section was considered as a new seed section with its own mean and standard deviation. The current section under evaluation was also closed whenever the database showed changes in the motorway identification code and in the direction of travel. This process was implemented repeatedly along the network, with the resulting identification of 554 homogeneous sections having a length of at least 300 m.

The threshold values and reference length adopted for the segmentation procedure were selected as operational parameters for network-level analysis. The 300 m minimum length was chosen as a compromise between spatial resolution and statistical robustness, since the objective was not local defect detection at 20 m scale, but homogeneous-section evaluation for PAMS implementation. The 30% and 70% thresholds for mean and standard deviation were used to identify sections with comparable central tendency and variability. The DI > 2.5 filter was introduced to remove anomalous extreme values of the smart tire response before section aggregation.

In addition to its version based on the analysis of vehicle DI values, as described above, the sectioning method was implemented separately by considering left DI and right DI values, thereby creating other two sets of homogeneous road sections of the motorway network. Moreover, the method, in which the initial DI filter (>2.5) was omitted, was also utilized to create three further sets of homogeneous sections based on vehicle, left, or right Pr.

Finally, for each set of homogeneous sections resulting from the iterative process described above, the mean values of the various functional condition parameters in each homogeneous section were determined for subsequent use in correlation analysis.

Since Method A is based on the internal consistency of smart tire indicators, a possible segmentation-related bias cannot be excluded a priori. For this reason, an alternative segmentation strategy, referred to as “Method B,” was introduced. Unlike Method A, Method B identifies homogeneous motorway sections exclusively based on pavement-wearing course type. Thus, the database was initially sorted out by motorway, carriageway, pavement type and increasing chainage. Adjacent rows of data referring to elementary road sections of 20 m length with the same type of pavement surface layer were merged together and considered homogeneous, iteratively repeating the process until changes were identified with respect to motorway identification code or the direction of travel. Elementary road sections with DI greater than 2.5 were excluded as outliers, and homogeneous sections shorter than 300 m were discarded, thereby yielding a total of 200 homogeneous sections longer than 300 m. As in the case of Method A, for each homogeneous section, the mean values of the various functional condition parameters were considered for subsequent correlation analysis.

## 6. Results

### 6.1. Database Overview

Table 2 and Table 3 present summary statistics of the cleaned database, after outlier removal but before the identification of homogeneous sections, of the 405 km motorway network. These tables illustrate the distribution and variation across the network of the DI and Pr parameters, as well as of the IRI and MPD values, providing insight into the overall condition of the surveyed motorways.

According to Table 2, the vehicle Dynamic Index ranges from 0.02 to a maximum of 2.49, with a mean of 0.43 which is closer to the minimum. The 25th and 75th percentiles of vehicle DI are equal to 0.15 and 0.56, respectively, indicating that 50 percent of the values fall in this interquartile range. Similar trends with greater variance were observed for right and left DI values. IRI values range from 0.3 to 9.7 mm/m, reflecting the presence of a variety of roughness levels. The mean values of DI and IRI exceed their medians, confirming the existence of right-skewed distributions. The mean value of the average IRI (computed by considering the longitudinal profiles recorded along the two wheel tracks), equal to 1.36 mm/m, indicates that most surveyed pavements were in good conditions, with no major occurrence of differential settlements or uneven deformation of asphalt layers.

As shown in Table 3, vehicle Pr is comprised between −0.11 and 1.27, with a mean value equal to 0.57. The 25th and 75th percentiles are equal to 0.43 and 0.73, respectively, while the standard deviation of 0.21 corresponds to one third of the mean. Similar statistical parameters were found for left and right Pr. MPD ranges from 0.3 to 2.7 mm, indicating the presence of a wide variety of macrotextures. Pr values were shown to follow a normal distribution, while MPD exhibited left skewness due to the predominance of two types of wearing courses (dense-graded asphalt, with a lower MPD, and open-graded asphalt, with a higher MPD). The mean value of the average MPD, equal to 1.2 mm, confirms that most surveyed pavements were in good condition, with no significant evidence of ongoing deterioration or raveling phenomena.

When focusing the analysis only on the data coming from smart tires, it was observed that DI values were much more scattered than Pr data, with the presence of a higher number of outliers. Furthermore, relatively small differences were recorded between left and right Pr values. Such an outcome is consistent with expectations, since pavement macrotexture is typically similar along the left and right wheel tracks, and evolves similarly in time as a result of vehicle loading. As expected, greater differences were identified when comparing the left and right DI, since they were obtained from pairs of smart tires which follow distinct road profiles.

### 6.2. Correlations Between Smart Tire Data and Reference Data

To investigate the existence of correlations between smart tire data and results obtained by means of the ARAN vehicle, analyses were carried out by referring to the homogeneous sections identified by using the previously described two alternative approaches (i.e., Method A and Method B).

**Figure 1:**
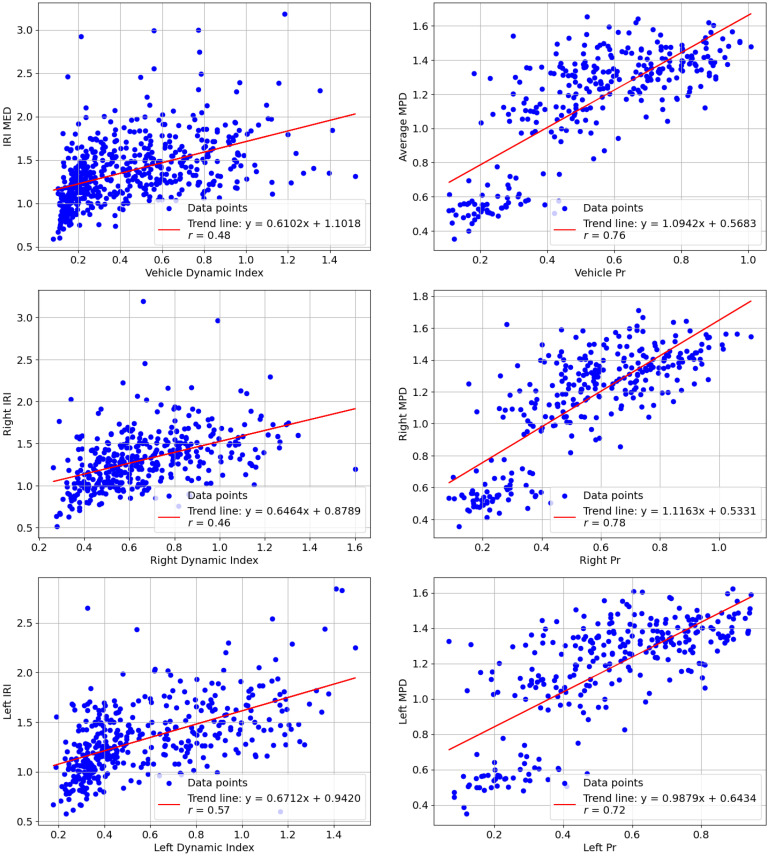
Correlations between smart tire and ARAN indicators (sectioning method A).

Correlations obtained while considering homogeneous sections obtained by means of Method A (i.e., by utilizing criteria based on consistency of smart tire data) are displayed in Figure 1. In general terms, it can be observed that, as expected, an increase in DI corresponds to an increase in IRI, and an increase in Pr corresponds to an increase in MPD. Such an outcome confirms the existence of a direct relationship between indicators drawn from smart tires and those obtained by means of the more classical surveying technique. In other words, higher DI correctly reflects the presence of rougher profiles, and higher Pr indicates coarser pavement macrotexture. According to the highlighted Pearson coefficients (r), DI exhibits a moderate positive correlation with IRI (r of the order of 0.5–0.6), while Pr demonstrates a strong positive correlation with MPD (r = 0.7–0.8). Parameters pertaining to the entire vehicle (i.e., by considering the entire set of smart tires) or to pairs of tires (i.e., left and right) show similar trends. It should be mentioned that in Pr versus MPD plots, the clearly visible lower-left cluster corresponds to pavements with a dense-graded asphalt-wearing course, which are distinctly separated from the upper-right cluster associated with pavements with open-graded asphalt surface course.

**Figure 2:**
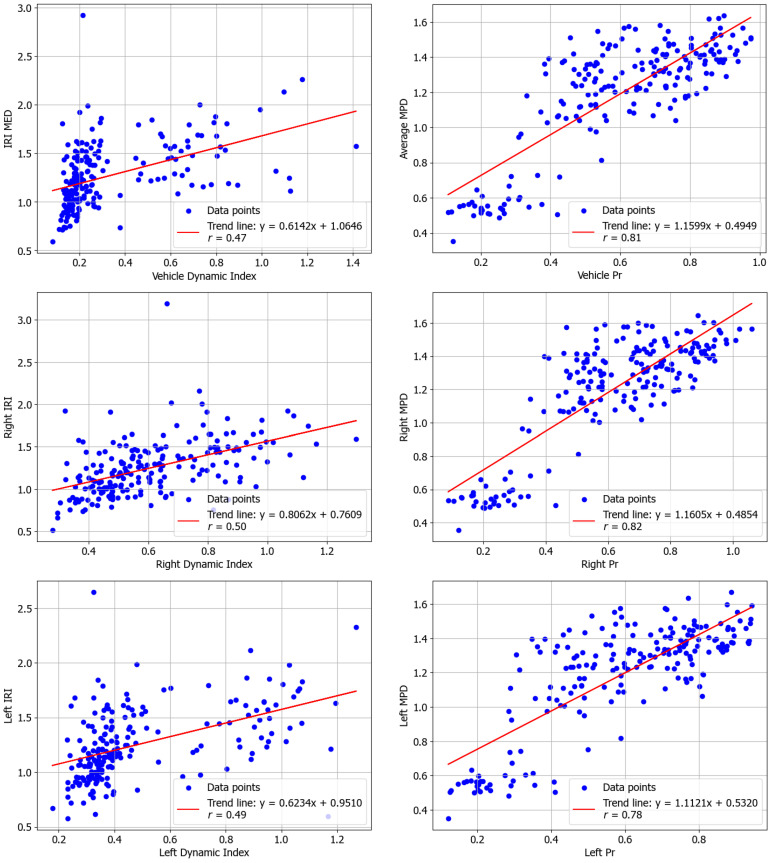
Correlations between smart tire and ARAN indicators (sectioning method B).

Correlations identified while referring to the homogeneous sections generated by means of Method B (i.e., by focusing exclusively on wearing course type) are shown in Figure 2. Displayed trends confirm the previously described findings synthesized in Figure 1 and demonstrate that the second sectioning method leads to correlations which are equivalent to those associated with the first one, revealing for both pairs of indicators the existence of moderate-to-very-strong relationships (r of approximately 0.5 for DI and IRI, r of the order of 0.8 for Pr and MPD). The consistency of the results obtained using the two independent sectioning approaches supports the robustness of the observed relationships and reduces the likelihood that the reported correlations are an artifact of the segmentation procedure.

It should be noted that the reported correlations refer to homogeneous-section averages and not to the original 20 m elementary records. Aggregation reduces local variability and is therefore expected to produce clearer relationships than point-to-point comparisons. Consequently, the obtained coefficients should be interpreted at the network/homogeneous-section scale, which is the target scale of PAMS implementation, and not as evidence of local equivalence between smart tire indicators and ARAN measurements. In addition, it should be mentioned that the correlation analysis of data generated by means of the two considered homogenization methods was also performed after filtering out data points collected in the correspondence of bridges and viaducts. However, no significant changes were observed with respect to the general trends and to the strength of identified correlations.

Detailed statistical parameters of the regression analyses between the smart tire and ARAN indicators presented in Figure 1 and Figure 2 are provided in Table 4. For all regression models, the *p*-value is less than 0.001, indicating that the observed relationships are highly statistically significant. The lower R^2^ values for DI vs. IRI correlations observed in the present study compared to Montorio et al. [25] are attributed to the broader motorway-scale dataset, wider variability of pavement types and operating conditions, different aggregation procedures, and the use of an extended real-network validation rather than a more controlled dataset.

### 6.3. Effect of Pavement Age on Smart Tire Indicators Data

In the context of pavement management systems, it is of premium importance to verify whether smart tire indicators can aid in identifying the time-dependent and traffic-dependent evolution of pavement roughness and macrotexture. Thus, the database created during the research project was subjected to further analyses in order to identify specific trends pertaining to this aspect. For such a purpose, as illustrated in the following, another sectioning method was introduced in the data processing.

While considering the overall database referred to elementary road sections of 20 m length, data strings (or rows) with a vehicle DI greater than 2.5 were initially removed as outliers. Such preliminary filtering was implemented only when carrying out analyses focused on DI values, whereas it was omitted when considering Pr data. Following the preliminary phase of data cleaning (where applicable), each row was then assigned a label based on its pavement age. For example, all data collected in the correspondence of pavements with ages of less than 1 year were associated with age class “0–1”, all rows with pavement ages between 1 and 2 years were assigned to age class “1–2”, and so on. Furthermore, since the majority of pavements surveyed in the network had dense-graded (46 km) or open-graded (331 km) wearing courses, only the indicators associated with these types of surface layers were retained in the database for further analysis. As in the case of the homogenization methods (A and B) adopted for the identification of general correlations, such database reduction operations were repeated for all the considered smart tire indicators, thereby leading to six distinct databases for each wearing course type. Age-dependent trends were thereafter investigated separately for the two types of wearing courses and by considering, for comparative reasons, the functional condition indicators drawn from the smart tires and those coming from the ARAN vehicle. In such a context, reference was made, for each age class, to the median values of DI and to the average values of all other indicators.

Preliminary analyses showed that simple linear correlations between pavement age and all indicators are relatively weak and characterized by a limited statistical significance. Such an outcome can be explained by considering that the progressive evolution of pavement roughness and macrotexture is dependent not only on traffic loading and environmental exposure, but also on other factors that include, among others, overall pavement structural behavior, construction quality, the occurrence of site-specific loading conditions, and the execution of localized maintenance and repair works.

As a result of the above, obtained results were subjected to further analyses in which the issue of roughness and macrotexture evolution was tackled by following a holistic and more pragmatic approach. In particular, rather than referring to the results associated with all age classes, indicators were computed for four characteristic periods, indicated as “post-construction”, “early life”, “stationary”, and “degradation”. As shown in Table 5, for each wearing course type, the age classes corresponding to the various characteristics periods were selected to meaningfully match experimental results.

**Figure 3:**
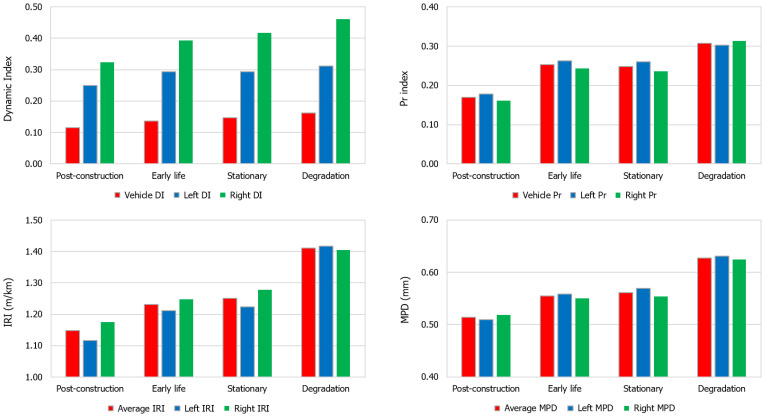
Time-dependent trends of smart tire and ARAN indicators (dense-graded wearing courses).

**Figure 4:**
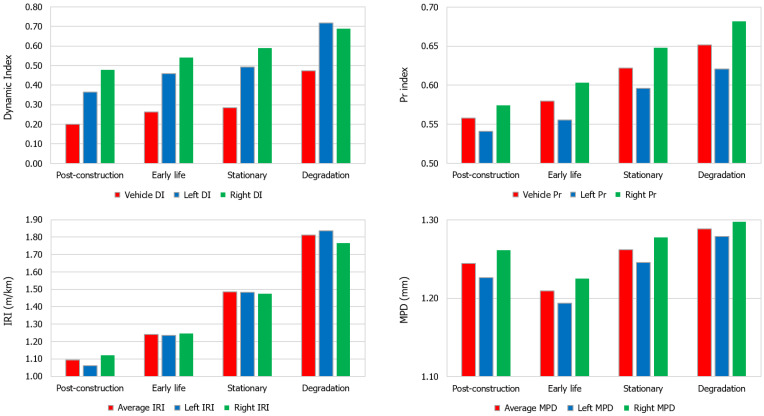
Time-dependent trends of smart tire and ARAN indicators (open-graded wearing courses).

Results obtained by following the approach outlined above are shown in the bar charts of Figure 3 (dense-graded wearing courses) and Figure 4 (open-graded wearing courses). Represented values of the functional condition indicators were computed from the median or average values of each age class, thereafter averaged across each considered period.

From the data represented in Figure 3 and Figure 4, it can be observed that all considered indicators displayed a progressive increase throughout wearing course service life, with trends that are similar for parameters drawn from smart tires and from the ARAN vehicle. The only exception is represented by MPD measurements performed on open-graded wearing courses, which exhibited a slight decrease in the “early life” period, which is not captured by the parameter drawn from the analysis of smart tire acceleration signals. Nevertheless, displayed data indicate that, within a PAMS, thresholds can be established with respect to any of the considered indicators to identify the need for maintenance works, or to select road sections that need to prioritized in such respect. When examining the bar charts more closely, it is interesting to note that in each characteristic period, average values of the vehicle DI are significantly lower than those of the left and right DI, thereby indicating that intervention thresholds need to be appropriately tailored depending upon the considered parameter.

### 6.4. Smart Tire Data for Road Network Management

As highlighted in the introduction, one of the main goals of a PAMS is to establish priorities for budget allocation and the execution of maintenance works. Thus, when considering a vast motorway network, such as the one investigated in this paper, it is essential to identify meaningful functional condition rankings of the various component segments. This activity can be extremely challenging and in such a context, it is of paramount importance to verify whether the data collected by means of smart tire technology can replace those coming from the more cumbersome traditional monitoring techniques.

Relative rankings obtained by considering the data collected on the motorway segments subjected to investigation are displayed in Table 6 and Table 7, which also show the mean values of the various functional condition indicators drawn from the processed database. The higher rankings are associated with the higher values of the indicators, thereby highlighting a higher priority for budget allocation and intervention. Displayed IRI and MPD values are the averages derived from the longitudinal profile of both wheel tracks, while DI and Pr values are those computed from the signals of all smart tires (i.e., they are “vehicle” indicators).

It can be observed that rankings assigned by referring to ARAN indicators and to smart tire indicators were consistent. This result supports the use of smart tire data for high-level network screening and prioritization within PAMS.

## 7. Ongoing Research Work

Although the results discussed in the previous section of this paper can be considered promising, it should be mentioned that since the completion of the research project, further work has already been initiated and is in progress on a wider set of experimental data including smart tire indicators and more traditional roughness and macrotexture parameters. Such activities are being carried out with the purpose of fine-tuning obtained correlations and of consequently increasing their reliability. Preliminary outcomes suggest that obtained correlation coefficients exceed those provided in this paper, thereby providing further support with respect to the possibility of fully implementing the smart tire technology in currently available network-level PAMSs.

## 8. Conclusions

The research work presented in this paper focused on the use of the “smart tire”— or “intelligent tire”—technology for the evaluation of pavement roughness and macrotexture along several segments of a motorway network. The objective of the study was to assess the suitability of smart tire indicators, namely Dynamic Index (DI) and Pr index, for supporting pavement asset management systems through comparison with standard ARAN-derived parameters, namely IRI and MPD.

Obtained results, based on a specific data processing technique employed for the identification of homogeneous sections, indicate that moderate correlations exist between DI and IRI, while stronger correlations were observed between Pr and MPD.

Pavement-age analyses showed that smart tire indicators are able to reproduce broad time-dependent trends that are consistent with those derived from conventional measurements. Moreover, the ranking of motorway segments based on smart tire indicators was consistent with the ranking obtained from IRI and MPD, suggesting that the technology can be useful for network-level screening and prioritization.

Based on the above, it can be concluded that the smart tire technology is sufficiently developed and mature to be regarded as a promising complementary monitoring technology for frequent, large-scale PAMS-oriented assessment, thereby providing a real-time quantitative evaluation of pavement functional conditions. Ongoing research work also demonstrates that such a technology is quickly progressing further, with the possibility of significantly increasing its reliability. However, conventional reference surveys remain necessary for calibration, validation, and project-level decisions.

Future research work will include a more detailed analysis of the effects associated with the various factors that influence the evolution of pavement roughness and macrotexture, and will also consider the possibility of implementing machine learning algorithms.

## Figures and Tables

**Table 1:** Surveyed motorway segments.

Motorway ID	Surveyed Extension (km)
ID-1	1.220
ID-2	98.400
ID-3	184.400
ID-4	86.900
ID-5	35.800

**Table 2:** Summary statistics of IRI and DI over the surveyed motorway network.

	Left IRI	Right IRI	Average IRI	Left DI	Right DI	Vehicle DI
Mean	1.36	1.34	1.36	0.64	0.70	0.43
Standard deviation	0.75	0.70	0.69	0.46	0.43	0.43
Skewness	2.71	2.48	2.57	1.51	1.49	2.10
Minimum	0.29	0.34	0.37	0.05	0.07	0.02
25th percentile	0.90	0.89	0.92	0.31	0.40	0.15
50th percentile	1.17	1.16	1.18	0.49	0.58	0.28
75th percentile	1.59	1.56	1.57	0.84	0.88	0.56
Maximum	9.35	9.72	9.57	2.50	2.50	2.49

**Table 3:** Summary statistics of MPD and Pr over the surveyed motorway network.

	Left MPD	Right MPD	Average MPD	Left Pr	Right Pr	Vehicle Pr
Mean	1.20	1.21	1.21	0.55	0.60	0.57
Standard deviation	0.29	0.30	0.29	0.22	0.23	0.21
Skewness	−0.80	−0.72	−0.85	0.14	0.11	0.06
Minimum	0.31	0.32	0.31	−0.17	−0.05	−0.11
25th percentile	1.06	1.08	1.08	0.39	0.45	0.43
50th percentile	1.25	1.26	1.26	0.53	0.59	0.56
75th percentile	1.39	1.41	1.40	0.72	0.75	0.73
Maximum	2.22	2.71	2.23	1.33	2.06	1.27

**Table 4:** Statistical parameters of the regression analyses between smart tire and ARAN indicators.

	Related Graph	95% Conf. Interval	R^2^	RMSE
Method A	IRI MED vs. Vehicle DI	[0.516, 0.704]	0.23	0.32
	Right IRI vs. Right DI	[0.523, 0.770]	0.21	0.30
	Left IRI vs. Left DI	[0.576, 0.767]	0.32	0.29
	Average MPD vs. Vehicle Pr	[0.986, 1.203]	0.57	0.21
	Right MPD vs. Right Pr	[1.012, 1.221]	0.60	0.21
	Left MPD vs. Left Pr	[0.875, 1.101]	0.51	0.22
Method B	IRI MED vs. Vehicle DI	[0.455, 0.774]	0.22	0.29
	Right IRI vs. Right DI	[0.614, 0.999]	0.25	0.28
	Left IRI vs. Left DI	[0.466, 0.781]	0.23	0.29
	Average MPD vs. Vehicle Pr	[1.041, 1.278]	0.66	0.19
	Right MPD vs. Right Pr	[1.046, 1.275]	0.67	0.19
	Left MPD vs. Left Pr	[0.987, 1.237]	0.61	0.20

**Table 5:** Characteristic periods for the analysis of the time-dependent evolution of pavement roughness and texture.

Characteristic Period	Pavement-Age Classes			
	Dense-Graded Wearing Course	Number of Data Points	Open-Graded Wearing Course	Number of Data Points
Post-construction	from 0–1 to 1–2	235	from 0–1 to 1–2	1151
Early life	from 1–2 to 4–5	660	from 1–2 to 4–5	3771
Stationary	from 4–5 to 9–10	827	from 4–5 to 15–16	7358
Degradation	from 9–10 to 12–13	592	from 15–16 to 18–19	4276

**Table 6:** Ranking of motorway segments based on smart tire and ARAN pavement roughness indicators.

Motorway ID	Mean IRI	IRI Ranking	Mean DI	DI Ranking
ID-5	1.31	1	0.55	1
ID-3	1.28	2	0.53	2
ID-1	1.14	3	0.37	3
ID-4	1.09	4	0.16	4
ID-2	0.95	5	0.15	5

**Table 7:** Ranking of motorway segments based on smart tire and ARAN pavement macrotexture indicators.

Motorway ID	Mean MPD	MPD Ranking	Mean Pr	Pr Ranking
ID-2	1.37	1	0.81	1
ID-4	1.33	2	0.73	2
ID-1	1.28	3	0.71	3
ID-3	1.22	4	0.50	4
ID-5	1.21	5	0.49	5

## Data Availability

Restrictions apply to the collected datasets.

## Abbreviations

The following abbreviations are used in this manuscript:

AASHTO	American association of state highway and transportation officials
ARAN	Automatic road analyser
ASCII	American standard code for information interchange
ASTM	American society for testing and materials
BS	British Standard
Coh	Coherence
Conf.	Confidence
DI	Dynamic Index
EN	European norm
EqPSD	Equivalent power spectral density
FFT	Fast Fourier transform
GPS	Global positioning system
ID	Identification code
in	Inch
IRI	International roughness index
ISO	International organization for standardization
m	Meter
mi	Mile
kHz	Kilohertz
km	Kilometer
MEMS	Micro-electro-mechanical system
MFV	Multi-functional vehicle
mm	Millimeter
MPD	Mean profile depth
μm	Micrometer
NSV	Network survey vehicle
PAMS	Pavement asset management system
PIARC	Permanent international association of road congress
Pr	Pr index
PSD	Power spectral density
r	Pearson correlation coefficient
R^2^	Coefficient of determination
RMS	Root mean square
RMSE	Root mean squared error
ROMDAS	Road measurement data acquisition system
RST	Road Surface Tester
SAW	Surface acoustic wave
VEU	Vehicle elaboration unit
VRR	Vehicle radio receiver
